# Supplementary material for: The Dual Prey-Inactivation Strategy of Spiders—In-Depth Venomic Analysis of Cupiennius salei
Source: Toxins (Basel). 2019 Mar 19;11(3):167. doi: 10.3390/toxins11030167 (PMC6468893; doi:10.3390/toxins11030167)
Supplement: Supplementary file 1 [file toxins-11-00167-s001.zip › Supplementary Fig S6/Supplementary Fig S6.pdf]

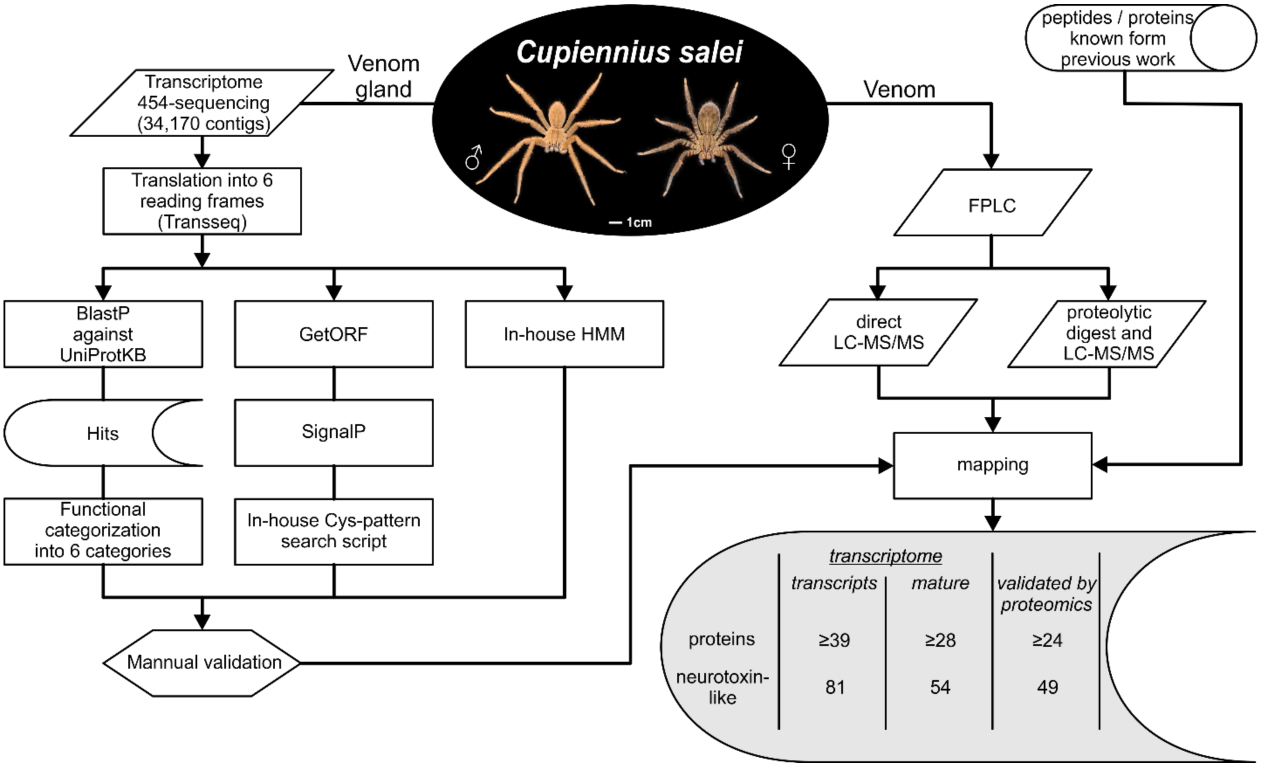

Supplementary Fig S6. Venomics workflow.

Flowchart detailing the analyses of the *C. salei* venom gland transcriptome (left side) and proteome (right side).
